# Performance of a New Tabletop Non-mydriatic Fundus Camera for Single-Field Diabetic Retinopathy Detection: A Pilot Study

**DOI:** 10.7759/cureus.88198

**Published:** 2025-07-17

**Authors:** Winston Padua, Shalini Butola, Suneetha Nithyanandam, Tony Raj, Dhinagaran D, Lavanya Chidambara, Lakshmi Krupa, Satish Bhandarkar, Aparna Gunda

**Affiliations:** 1 Opthalmology, St. John’s Medical College Hospital, Bengaluru, IND; 2 Opthalmology, St. John’s Research Institute, Bengaluru, IND; 3 Opthalmology, Sapthagiri Institute of Medical Sciences and Research Centre, Bengaluru, IND; 4 Opthalmology, Oivi Tech, Bengaluru, IND; 5 Clinical Research, Oivi Tech, Bengaluru, IND

**Keywords:** diabetic retinopathy detection, diabetic retinopathy screening, non-mydriatic fundus camera, tabletop fundus camera, topcon nw400

## Abstract

Introduction: Diabetic retinopathy (DR) detection is made easy with the use of a fundus camera. The evidence of the use of a fundus camera for DR detection in non-mydriatic conditions with limited technical challenges is scarce. This is a pilot study that evaluates the performance of the Oivi fundus camera (Oivi AS, Oslo, Norway), a novel non-mydriatic tabletop fundus camera for DR detection using a single-field, macula-centered imaging approach. Its diagnostic accuracy was compared with that of a standard reference device, the Topcon NW400 fundus camera (Topcon Corporation, Tokyo, Japan).

Methods: A total of 243 subjects with diabetes mellitus (DM) were recruited. Non-mydriatic macula-centered images were captured using both cameras. Two ophthalmologists independently graded the deidentified images for image quality and DR stage. Discrepancies between their assessments were adjudicated by consensus after review by a senior ophthalmologist. The senior ophthalmologist's grading of images from the standard camera images served as the ground truth for comparative analysis. Inter-modality agreement was evaluated using linear weighted kappa (κ) correlation.

Results: DR was detected in 23% of patients using the standard reference camera (12% of eyes) and in 23.86% using the tabletop camera (12.6%). Identification of moderate non-proliferative diabetic retinopathy (NPDR) (7.2%), severe NPDR (0.4%), and proliferative diabetic retinopathy (PDR) (1.23%) was similar between the two cameras, although not always in the same eyes. The inter-modality agreement (k) for DR was 0.927 (95% CI: 0.88-0.97) (almost perfect). The tabletop camera showed a sensitivity of 92.98% (95% CI, 83-98.05%) and a specificity of 99.47% (95% CI, 98.10-99.94%) for DR. The percentage of usable images was 92.3% with the standard reference camera and 95.2% with the tabletop camera.

Conclusions: This study provides preliminary evidence that the novel tabletop Oivi fundus camera may offer comparable performance to standard non-mydriatic devices for DR detection in a single-field strategy. Its portability and usability under mesopic conditions suggest potential value for point-of-care screening. Further large-scale studies are warranted to validate these findings and explore their role in screening programs.

## Introduction

Undetected diabetic retinopathy (DR) is the leading cause of vision impairment among individuals aged between 20 and 74 years [1,2]. Timely diagnosis of DR can also prevent systemic complications such as cardiovascular diseases, diabetic neuropathy, and nephropathy [3]. Successful DR screening programs have been shown to significantly reduce rates of vision loss [4, 5]. Furthermore, screening rates have improved from 35%-55% to 80%-85% when screening services are shifted from ophthalmologist clinics to primary care settings [6]. Despite these advances, the implementation of national-level diabetic eye screening programs remains inefficient and incomplete, particularly in lower-middle-income countries and many higher-middle-income and high-income countries [7].

Integrating telemedicine solutions with rapid, non-mydriatic retinal imaging systems that require minimal operator expertise is essential for scaling DR screening efforts [8]. This is especially important in resource-constrained settings where affordability and ease of use are necessary for widespread adoption. Based on level I evidence, the American Academy of Ophthalmology guidelines recommend the use of single-field 45-degree fundus photography interpreted by trained graders for DR detection [9]. The advantages of this approach are that it takes less time, and it is convenient to the patient as it uses only one flash of light to image. More importantly, it may be a cost-effective way of using ophthalmic services, as only patients with vision-threatening DR are referred to an ophthalmologist, and other DR severity levels can be deferred. This will be effective, especially in the regions where the number of ophthalmologists is limited, and aligns well with the large-scale screening protocols [9].

Considering the image non-gradability in non-mydriatic conditions, American Academy of Ophthalmology guidelines highlight that sensitivity and specificity improve with mydriasis [9]. However, evidence demonstrating that non-mydriatic cameras can achieve high diagnostic accuracy while minimizing technical challenges remains limited.

This study evaluated the performance of the Oivi fundus camera (Oivi AS, Oslo, Norway), a novel, tabletop, non-mydriatic imaging device, in comparison with the Topcon NW400 (Topcon Corporation, Tokyo, Japan), a widely used fundus camera. The objective is to validate the Oivi camera for detecting various severity levels of DR under non-mydriatic conditions, using a single, macula-centered image against the Topcon NW400 as the reference standard. The findings aim to establish the diagnostic accuracy of the Oivi camera and its potential utility for DR detection.

## Materials and methods

Study design

This is a single-center, prospective, observational, non-interventional, cross-sectional instrument validation study designed to evaluate the diagnostic performance of the Oivi fundus camera in detecting DR, using the Topcon NM400 fundus camera as the reference standard.

Oivi fundus camera

The Oivi camera is a tabletop, portable, non-mydriatic fundus imaging device. The system captures retinal images at 11.5 megapixels, with a field of view of 48 × 40 degrees and a working distance of 26 to 30 mm. An integrated pupil measurement module displays the pupil diameter on the corner of each image, enabling real-time assessment of pupil size. These pupil size measurements are not done manually but facilitated by an automated module that is integrated into the Oivi camera. A specially engineered plastic face mask enables imaging in ambient light conditions. This mask shields the eyes from light, inducing physiological mydriasis and facilitating imaging of small pupils (<4 mm) without needing pharmacological dilation. The image capturing process is easy and quick and takes a few minutes to capture four images per individual, images in two fields per eye. In the real world, the Oivi camera is enabled with automatic upload of images onto an online platform, facilitating remote grading for telemedicine applications in primary care and decentralized settings.

Topcon NW400 fundus camera

**Figure 1:**
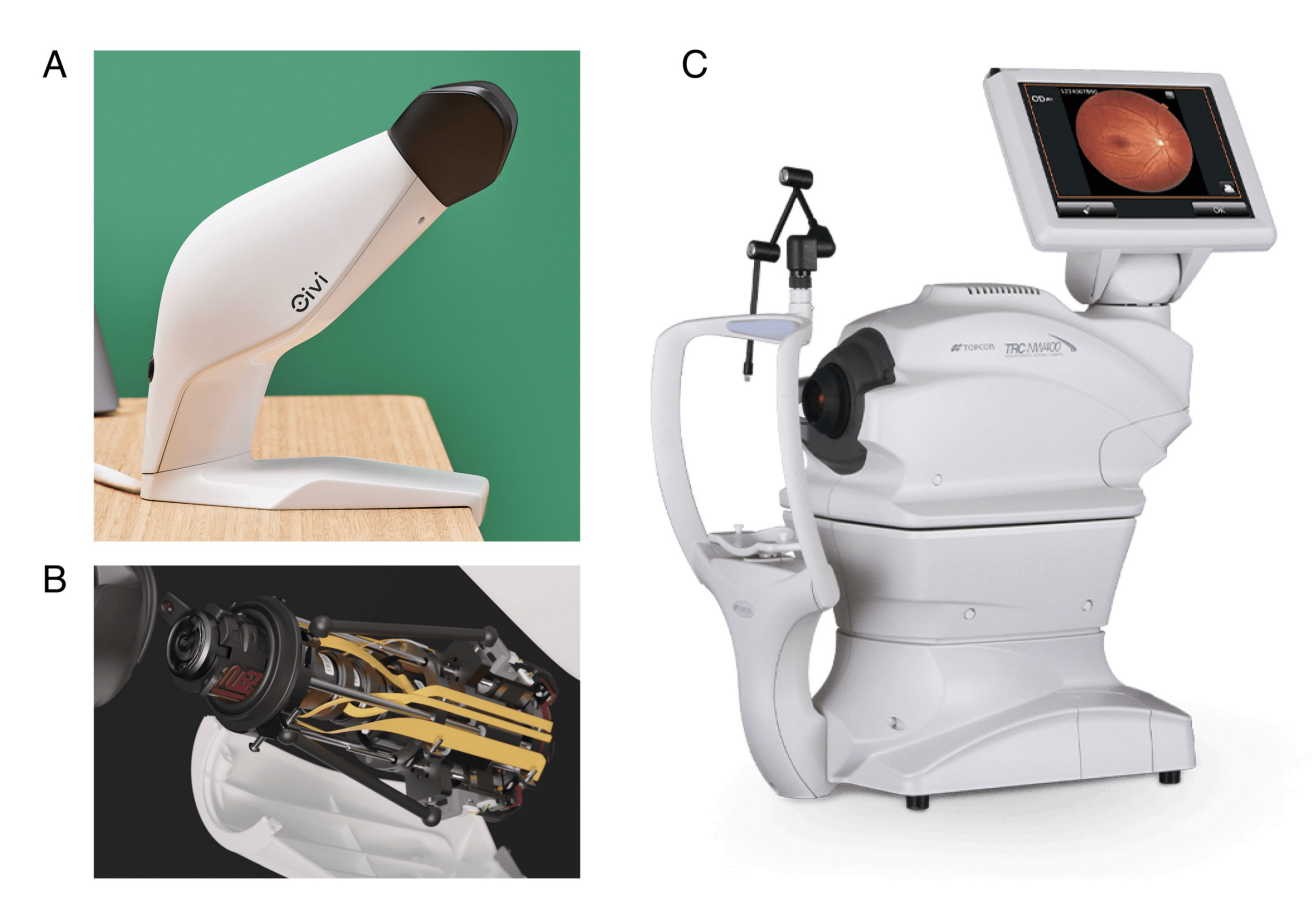
Images of the Oivi camera (A, B) and Topcon TRC NW400 camera (C)

The Topcon NW400 is a tabletop, non-mydriatic fundus camera that captures images with a 45-degree field of view and a working distance of 34.8 mm.​The device operates optimally with a pupil diameter of 4.0 mm or more and includes a small pupil mode that allows imaging with smaller pupils. Notably, Topcon’s small pupil mode was not utilised in our study. Imaging with this device requires a dark or dim-light environment. ​Images of both devices are provided (Figure 1).

Ethics approval and participant recruitment

This observational study was approved by the Ethics Committee (EC) of St. John’s Medical College Hospital, Bengaluru, India (IEC Study Ref. No. CT-20/2021). It adhered to the ethical principles of the 1964 Declaration of Helsinki. All participants provided written informed consent to participate in the study and to the use of the anonymized data for a publication.

Eligible participants were aged 18 years or older with confirmed type 1 or type 2 diabetes mellitus (DM). Exclusion criteria included patients with persistent vision loss, floaters, cataracts, corneal opacities, photosensitivity, or pregnancy. Between September and December 2022, 243 consecutive eligible patients were recruited.

Sample size estimation

We did not conduct any pilot study earlier to collect data on the agreement between the two fundus cameras. The sample size was estimated assuming that a moderate agreement would prevail between the two cameras. The sample size was estimated considering a DR prevalence of 20% (slightly higher than the prevalence of 12% in India) to accommodate a decent number of DR subjects for this comparative study. Sample size was estimated at 80% power to detect a linear weighted kappa (κ) of 0.8 versus a null hypothesis of κ=0.6 (moderate agreement vs. substantial agreement between two modalities), a two-tailed alpha of 0.05, and accounting for 10% non-gradable images, the estimated sample size was 214 participants [10]. We recruited 243 patients to ensure sufficient power.

Imaging process

All patients underwent bilateral macula-centered, single-field fundus imaging using both devices on the same day under non-mydriatic conditions. Imaging was first performed with the Oivi camera under ambient room lighting, followed by the Topcon camera after dimming the room lights, as per its user manual. A single trained operator, proficient with both devices and blinded to study objectives, acquired all images. Only one image per eye per device was captured, with no repeat acquisitions. These images were uploaded onto an online platform. 

Image grading

Two retina specialists (with a minimum of 10 years of experience), masked to patient information and each other’s evaluations, independently graded all images. Both the graders received training from a senior ophthalmologist on the images from both cameras on aspects of image quality and DR severity classification. Due to the difference in hue of the images, the graders were not truly masked for the camera. Images were randomly assigned and interpreted for image quality, DR severity, and diabetic macular edema (DME). The graders were provided the links to these images, through which they accessed the images and viewed and interpreted them on computer screens that were of the same make and specifications. These images were manually graded for quality, and no automated software tools were used. Image quality was assessed based on the validated systems that were reported earlier [11, 12]. The images were assessed for the extent of clear retinal visibility, discernibility of vasculature, and clarity of the macula and optic disc. Agreement between the graders has been assessed using the linear weighted κ correlation coefficient (Appendix A). Discordant grades were adjudicated by a senior ophthalmologist. Based on these features, images were categorized as excellent, good, adequate, acceptable, or unusable (Table 1).

**Table 1:** Definitions of image quality categories

Category	Description of images
Excellent	Images with no artefacts, highly visible retinal vasculature with clarity at the macula and optic disc.
Good	More than 90% of the image is visible with clear vasculature and clarity at the macula and optic disc.
Adequate	More than 70% of the image is clear with visible level III vascular arches with clarity at the macula and optic disc.
Acceptable	Less than 70% of the image is visible with/without noticeable DR lesions with the clear optic disc (visible fine vessels on disc) and/or interpretable macula (with visible third-generation vessels).
Unusable	More than 70% of the image is not clear with the presence of artefacts, with no clear level III vascular arches and with blurred temporal arcades. Lacks clarity at the optic disc and macula and cannot be interpreted.

The severity of DR and DME was assessed using the international clinical classification for DR [13]. The International Clinical Diabetic Retinopathy (ICDR) scale provides a classification of five stages of DR: (i) no apparent retinopathy (no DR); (ii) mild non-proliferative diabetic retinopathy (NPDR); (iii) moderate NPDR; (iv) severe NPDR; and (v) proliferative diabetic retinopathy (PDR). Vision-threatening diabetic retinopathy (VTDR) was defined as the presence of severe NPDR, PDR, and/or DME. Agreement between two graders for different DR categories was assessed by the linear weighted κ correlation coefficient (Appendix B).

Statistical analysis and clinical relevance strengths

Agreement between the two graders and two cameras (modalities) for DR categorization was assessed via linear weighted κ statistics. The chi-squared test was used to compare proportions between various groups. Sensitivity and specificity for the Oivi camera for DR were calculated against the Topcon camera as a percentage, along with confidence intervals and P-value. All the statistical analyses were performed via MedCalc statistical software version 23.2.7 (MedCalc Software Ltd, Ostend, Belgium). A P-value of < 0.05 was considered significant.

## Results

A total of 243 patients were recruited for the study. A total of 485 eyes were analyzed, as only the right eye image of one patient was analyzed. Over half of the cohort had been diagnosed with type 2 DM for more than five years (53.9%), with a median duration of five years (0.1-25 years) (Table 2).

**Table 2:** Baseline characteristics of the patients enrolled in the study DM: diabetes mellitus; HT: hypertension

Variable		Percentage of total cohort (%)
Gender	Male	61.32
	Female	38.68
Age	<40 years	18.60
	40-60 years	63.71
	>60 years	17.31
	Median age in years (range)	51 (29-69)
Pupil size	Greater than 4mm	96.70
	Greater than 4.5mm	75.46
	Median pupil size (mm)	5.1 (3.3-7.3)
Medical history	DM: less than five years	46.09
	DM: above five years	53.91
	Median duration of DM in years (range)	5 (0.1-25)
	HT	50.60
	Median duration of HT in years (range)	5 (0.1-25)
	DM and HT > five years	21.40

Regarding the pupil diameter (as measured by the Oivi camera), 96.7% of patients had pupils greater than 4 mm, and 75.46% had pupils greater than 4.5 mm, with a median pupil diameter of 5.1 mm (3.3-7.3 mm).

Image quality assessment

**Figure 2:**
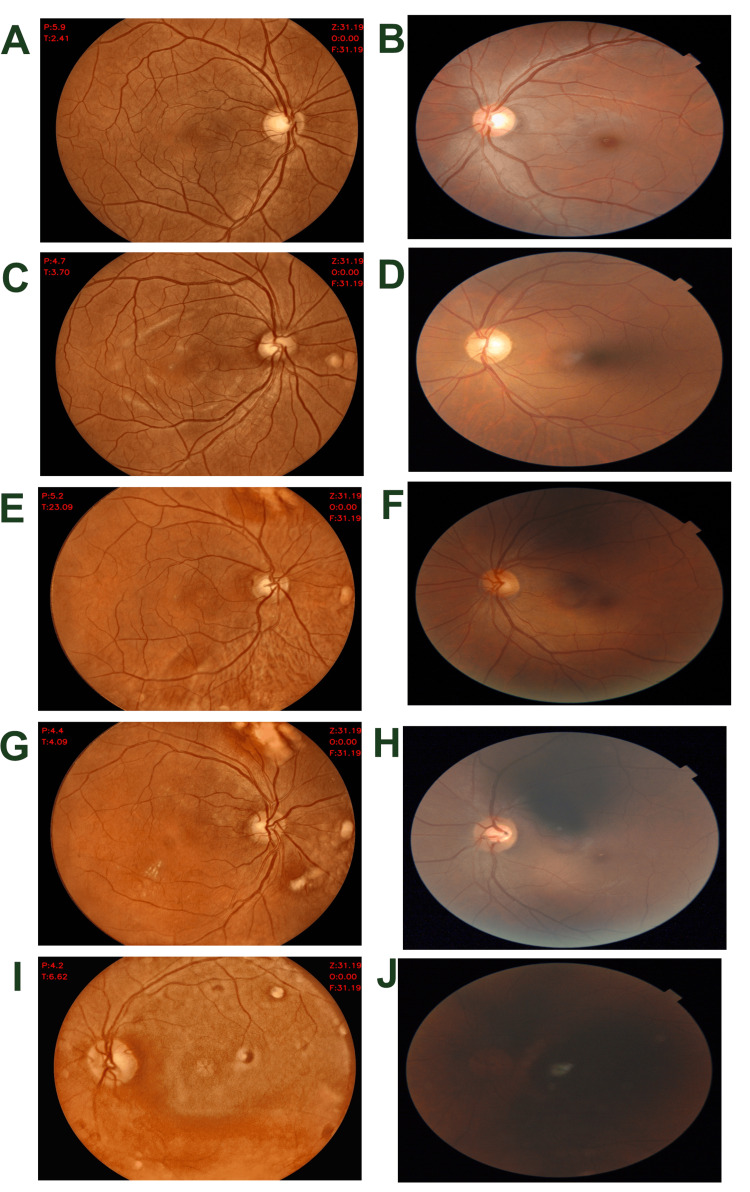
: Representative images of five image quality categories by both cameras Excellent by Oivi (A); good by Oivi (C); adequate by Oivi (E); acceptable by Oivi (G); unusable by Oivi (I); excellent by Topcon (B); good by Topcon (D); adequate by Topcon (F); acceptable by Topcon (H); unusable by Topcon (J)

Representative fundus images illustrating all five image quality categories for both Oivi and Topcon cameras are provided in Figure 2. Discrepancies in image quality categories were adjudicated by a senior ophthalmologist. Compared with Oivi, Topcon yielded a significantly (P<0.0001) higher percentage of excellent and acceptable images, whereas Oivi yielded a higher percentage (P<0.0001) of good-quality images. Adequate-quality images were similar between both cameras. Unusable images were more common with Topcon than with Oivi (P=0.03). When combining the top four quality categories into a single “gradable/usable” category (images suitable for DR diagnosis), 95.2% of Oivi images were usable compared to 92.3% of Topcon images. Image quality categories were further estimated in subgroups related to age and pupil size, which have a confounding effect on image quality. The Oivi camera produced a higher proportion of usable images in patients over 40 and 50 years of age (Table 3). In cases where pupil diameter was ≤4.5 mm, unusable images were significantly more frequent with Topcon than with Oivi (P = 0.03).

**Table 3:** Proportions of the five categories of images by both cameras

All eyes (n=485)	Camera	Excellent images	Good images	Adequate images	Acceptable images	Unusable images	Usable images
	Oivi	3.66	40.8	31	19.8	4.74	95.26
	Topcon	10.6	20.5	28.76	32.2	8.04	92.06
≥ 50 years (n=255)							
	Oivi	2.4	33.6	30.2	27.5	6.3	93.7
	Topcon	3.5	15.7	31	40	9.8	90.2
≥ 40 years (n=393)							
	Oivi	4.6	39.7	29.3	21.6	4.8	95.2
	Topcon	8.1	18.6	31.6	32.8	8.9	91.1
Pupils ≥ <4.5 mm (n=118)							
	Oivi	0.8	14.5	30.5	44.9	9.3	90.7
	Topcon	2.5	4.3	25	49.55	18.65	81.35

DR detection

DR was assessed in all the gradable/usable images that encompass the first four image quality categories, regardless of confounding factors for image quality, like age and pupil size. The unusable images were not used for DR assessment, as the image was obscured to form an opinion on DR. DR was detected in approximately 23% of individuals (23.86% by Oivi; 23% by Topcon) and 12% of eyes (12.6% by Oivi; 12% by Topcon). The detection rates for mild NPDR were slightly higher with Oivi (3.7%) compared to Topcon (3.09%). The rates for moderate NPDR (7.2%), severe NPDR (0.4%), and PDR (1.23%) were identical between cameras, although these DR stages were detected in different eyes by both cameras. DME was detected in a similar percentage of eyes by both cameras (Table 4).

**Table 4:** Identification of DR and DME represented by the number of eyes DR: diabetic retinopathy; DME: diabetic macular edema; CiDME: center-involving DME; NPDR: nonproliferative diabetic retinopathy; PDR: proliferative diabetic retinopathy

Condition	Oivi (%)	Topcon (%)
No DR	401 (82.6)	388 (80)
DR	61 (12.6)	58 (12)
Mild NPDR	18 (3.7)	15 (3.09)
Moderate NPDR	35 (7.2)	35 (7.2)
Severe NPDR	2 (0.4)	2 (0.4)
PDR	6 (1.23)	6 (1.23)
Inconclusive	23 (4.7)	39 (8.04)
No DME	405 (83.5)	414 (85.3)
DME	10 (2.06)	9 (1.85)
CiDME	3 (0.62)	4 (0.82)
Non CiDME	7 (1.44)	5 (1.03)
DME inconclusive	70 (14.43)	62 (12.78)

**Figure 3:**
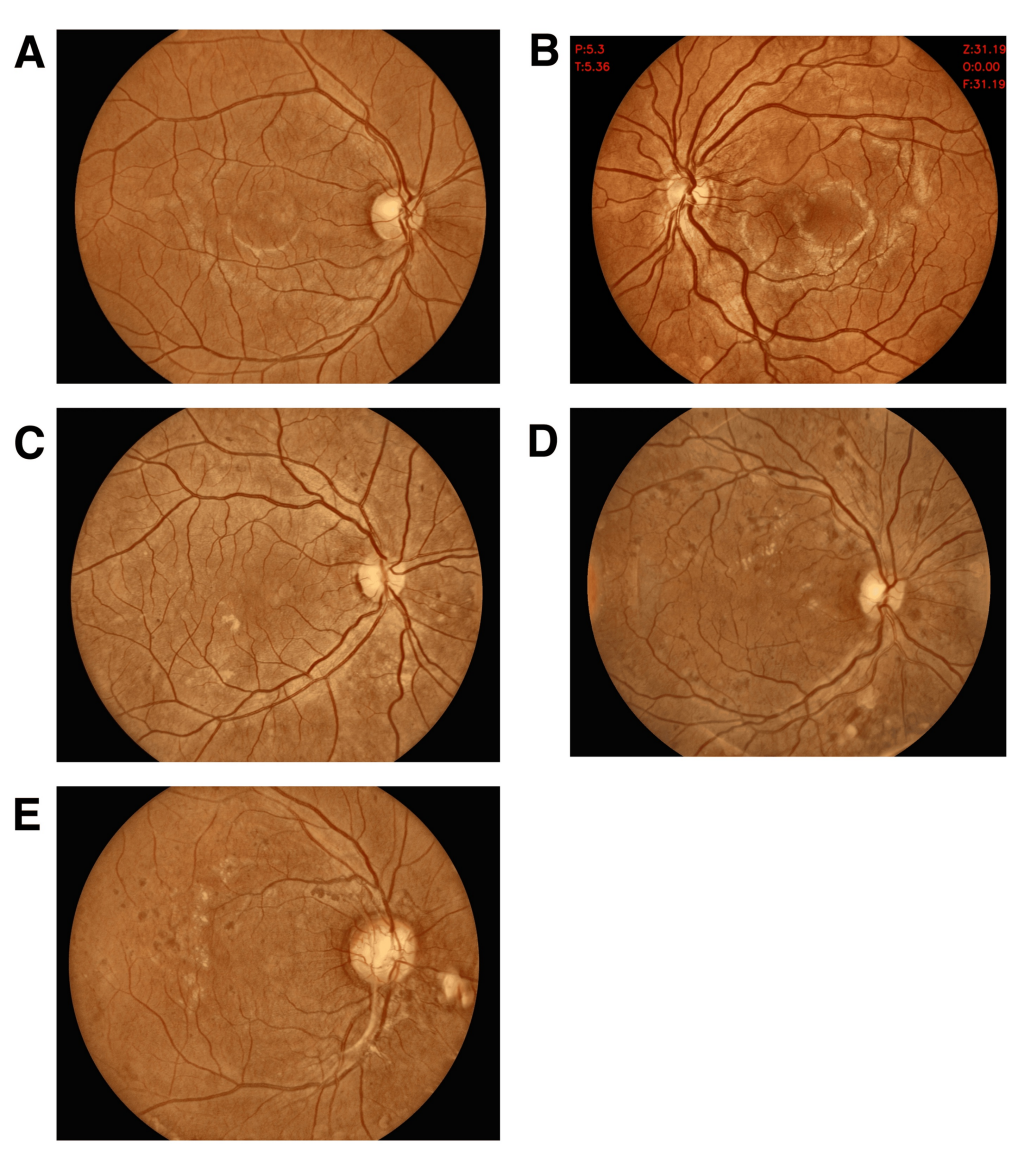
Representative images of no DR/normal retina and four severity levels of DR by the Oivi camera Normal retina with no DR (A), mild NPDR (B), moderate NPDR (C), severe NPDR (D), and PDR (E). DR: diabetic retinopathy; NPDR: non-proliferative diabetic retinopathy; PRD: proliferative diabetic retinopathy

**Figure 4:**
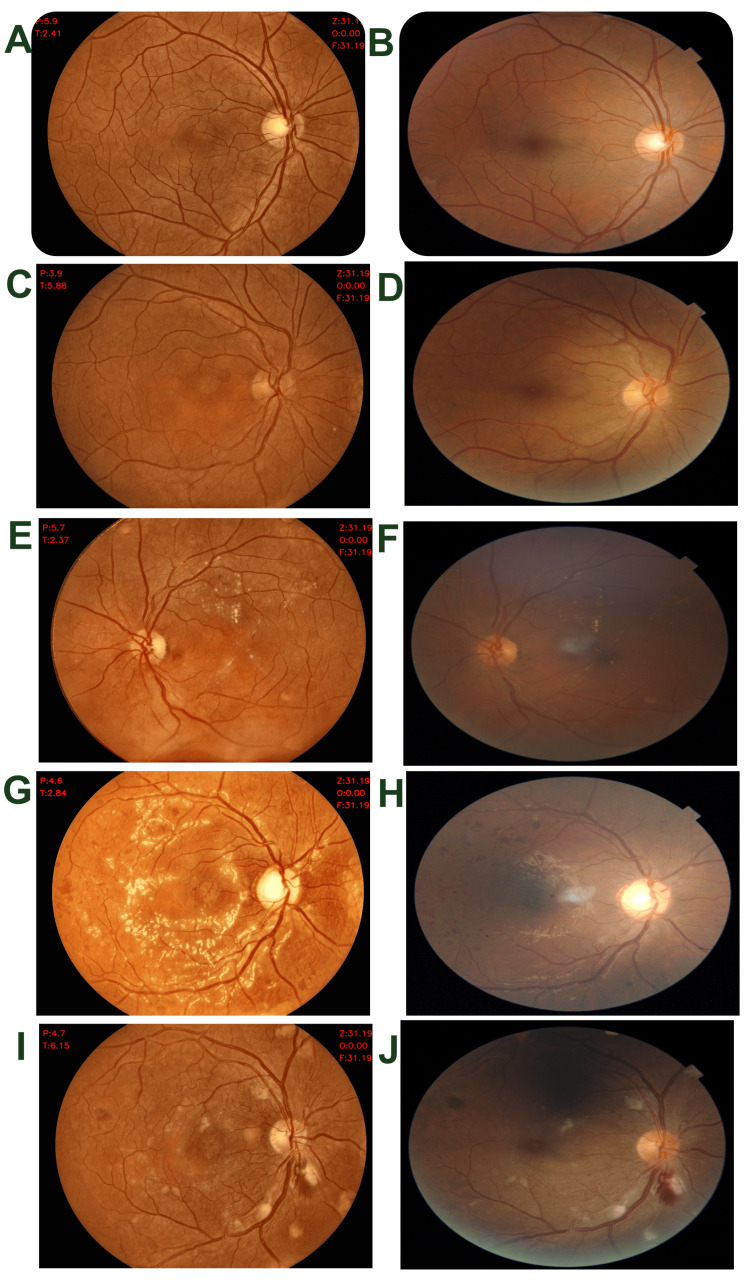
Images representing no DR and four severity levels of DR by Oivi and Topcon cameras on the same retina Normal retina with no DR by Oivi (A), mild NPDR by Oivi (C), moderate NPDR by Oivi (E), severe NPDR by Oivi (G), PDR by Oivi (I), normal retina with no DR by Topcon (B), mild NPDR by Topcon (D), moderate NPDR by Topcon (F), severe NPDR by Topcon (H), PDR by Topcon (J) DR: diabetic retinopathy; NPDR: non-proliferative diabetic retinopathy; PRD: proliferative diabetic retinopathy

The proportion of unusable or inconclusive images where DR staging could not be determined was significantly higher with Topcon (8.04% vs. 4.7%, P = 0.03). Examples of images representing no DR and the four stages of DR severity from the Oivi camera are shown in Figures 3A-3E, with paired images from both cameras provided in Figures 4A, 4C, 4E, 4G, 4I (Oivi camera) and Figures 4B, 4D, 4F, 4H, 4J (Topcon camera)).

Discrepancies between cameras included

Among 15 mild NPDR cases identified by Topcon, three were classified as no DR by Oivi. Among 18 mild NPDR cases identified by Oivi, two were classified as no DR and three as moderate NPDR by Topcon. For moderate NPDR, although the number of eyes was the same, classification differed: one Topcon moderate NPDR was PDR by Oivi, one was no DR, and three were mild NPDR by Oivi. Among 35 moderate NPDR cases identified by Oivi, four were inconclusive, and one was classified as PDR by Topcon. One PDR case identified by Topcon was inconclusive by Oivi, and vice versa. Notably, a total of 21 Topcon images that were deemed inconclusive were classified as no DR by the Oivi system, whereas only 10 Oivi-inconclusive images were identified as no DR by Topcon (Table 5).

**Table 5:** Grading of DR severity by two cameras DR: diabetic retinopathy; NPDR: non-proliferative diabetic retinopathy; PRD: proliferative diabetic retinopathy

	Topcon Camera							
	Condition	No DR	Mild NPDR	Moderate NPDR	Severe NPDR	PDR	Inconclusive images	Total
Oivi Camera	No DR	376	2	0	0	0	10	388
	Mild NPDR	3	12	0	0	0	0	15
	Moderate NPDR	1	3	30	0	1	0	35
	Severe NPDR	0	0	0	2	0	0	2
	PDR	0	0	1	0	4	1	6
	Inconclusive images	21	1	4	0	1	12	39
	Total	401	18	35	2	6	23	485

Comparative analysis

In eyes with gradable images from both cameras (n=435), absolute agreement exceeded 97.5% for any DR, referable DR, and vision-threatening DR, with almost perfect κ agreement (Table 6). The Oivi camera demonstrated a specificity of >99% for any DR and 100% for referable DR compared with Topcon. The sensitivity of Oivi was 92.98% (95% CI, 83.0%-98.5%) for any DR and 90.48% (95% CI, 77.38%-97.34%) for referrable DR (Table 6). However, the sensitivity and specificity for each grader are provided in Appendix B.

**Table 6:** Inter-modality agreement for DR and sensitivity and specificity CI: confidence interval; DR: diabetic retinopathy

DR category	Absolute agreement	Linear weighted kappa correlation (95% CI)	Kappa agreement
Any DR	97.50%	0.927 (0.88- 0.97)	Almost perfect
Referrable DR	99%	0.944 (0.89- 0.99)	Almost perfect
Vision-threatening DR	99.30%	0.838 (0.65-1)	Almost perfect
	Sensitivity (95% CI)	Specificity (95% CI)	
Any DR	92.98% (83.00% - 98.05%)	99.47% (98.10% - 99.94%)	
Referrable DR	90.48% (77.38% - 97.34%)	100% (99.07% - 100.00%)	

## Discussion

Effective screening tools that combine portability, ease of use, high image quality, and diagnostic accuracy are essential to improve the efficiency and impact of DR screening programs. Although many commercially available fundus cameras are designed to be user-friendly, comprehensive data on their performance, particularly in non-mydriatic conditions common in screening and in populations with Indian eyes, remains limited. The present study aims to address this gap. Our findings demonstrate that the image gradability and diagnostic accuracy of the non-mydriatic Oivi fundus camera are comparable to the universally approved Topcon camera, underscoring Oivi’s potential as a reliable and cost-effective tool for DR detection.

While indirect ophthalmoscopy and seven-field stereoscopic fundus photography of dilated fundus have historically been the gold standard for assessing retinal lesions, fundus photography is now widely accepted as a convenient and accurate method for DR detection [14]. Furthermore, studies confirm that monoscopic fundus images provide diagnostic equivalence to seven-field stereoscopic images for DR screening [9, 15].

Several prior studies have compared non-mydriatic fundus cameras against clinical examinations or seven-field early treatment diabetic retinopathy study (ETDRS) photography [16, 17] and established that macula-centered images are sufficient for DR detection and screening [18-20]. These studies have reported sensitivities between 38% and 100% and specificities between 76.5% and 99% for detecting any DR or referable DR using a single 45-degree image [15-17, 21, 22]. Non-gradability rates in these studies ranged from 4% to 26% [15-17, 21, 22]. However, few studies have focused on eyes of Asian or Indian participants, where darker irises may affect image quality. Limited studies from India and Sri Lanka, conducted in tertiary centers or community settings, reported sensitivities of 54.5% to 92.3% and specificities up to 96.8% for detecting any DR [23, 24], and up to 93% sensitivity and 90% specificity for vision-threatening DR [22]. Despite some clinicians advocating non-mydriatic cameras for DR screening, others have raised concerns over high nongradability rates (4%-43%) [24, 25]. In our study, the Oivi camera achieved >99% specificity for identifying patients without DR and demonstrated a sensitivity of 90.5%-93% for detecting DR, with a non-gradability rate of just 5%, aligning with international standards for DR screening programs [11, 26]. The study employed stringent gradability criteria focusing on the clarity of the optic disc, macula, and vasculature. While Topcon’s sensitivity has varied across populations and DR algorithms [27, 28], Oivi’s design-supporting physiological mydriasis may contribute to more consistent image quality, particularly in challenging cases involving smaller pupils.

Importantly, although this study did not use DR-artificial intelligence (AI) algorithms, integrating AI could offer further insights, particularly regarding the proportion of images that would require pupil dilation for automated DR detection, as recent studies have reported dilation in 78% of participants for AI-based DR diagnosis [28]. Nonetheless, the Oivi camera’s performance met international consensus thresholds of ≥80% sensitivity, ≥95% specificity, and <5% non-gradability [11, 26].

Given the rising prevalence of DM, especially among older adults, achieving high-quality images in patients over 50 years is critical for the success of DR screening programs [29]. In this group, the Oivi camera produced usable images in 93.7% of cases, 3.5% higher than Topcon camera. Among those over 60 and over 65 years, the rates of usable images were 89.3% and 81.6%, respectively (data not shown). The higher unusable image rate with Topcon camera may reflect limitations in its imaging technology when dealing with small pupils, whereas Oivi’s features likely mitigated this issue.

Limitations

Although the study matched the proportions of DR with the prevalence of DR in India, the study lacked a full spectrum of DR severity levels, particularly in vision-threatening DR. Furthermore, comparisons were limited to Topcon; inclusion of gold standard dilated fundus examination or seven-field ETDRS photography would provide a more comprehensive assessment of Oivi’s diagnostic accuracy by capturing peripheral lesions that would have been missed in the non-mydriatic conditions.

## Conclusions

The Oivi fundus camera demonstrates promising potential as a portable, non-mydriatic imaging device for DR detection, offering high-quality images with a high proportion of gradability. Its accuracy in identifying DR lesions is comparable to established reference standards, positioning it as a reliable and robust tool for screening applications. Further validation through large-scale, real-world community screening programs is warranted to fully establish its effectiveness and scalability as a frontline screening device.

## Appendices

Appendix A 

**Table 7:** Inter-camera and inter-observer agreement on five image quality categories vs.: versus; CI: confidence interval

Grader	Linear weighted kappa coefficient	95% CI	Kappa agreement
Grader 1: Oivi vs. Topcon	0.389	0.31-0.47	Fair
Grader 2: Oivi vs. Topcon	0.335	0.26-0.41	Fair
Oivi: Grader 1 vs. Grader 2	0.774	0.72-0.83	Substantial
Topcon: Grader 1 vs. Grader 2	0.722	0.66-0.77	Substantial

Appendix B 

**Table 8:** Inter-observer/modality agreement for DR and sensitivity and specificity DR: diabetic retinopathy; CI: confidence interval

Variable	Linear weighted kappa correlation coefficient (95% CI)	Kappa agreement
Inter-camera/modality agreement		
Grader 1-Any DR	0.946 (0.91 to 0.98)	Almost perfect
Grader 1-Referrable DR	0.944 (0.89 to 0.99)	Almost perfect
Grader 1- Vision-threatening DR	0.838 (0.66 to 1.0)	Almost perfect
Grader 2-Any DR	0.893 (0.84 to 0.95)	Almost perfect
Grader 2-Referrable DR	0.931 (0.87 to 0.99)	Almost perfect
Grader 2-Vision-threatening DR	0.745 (0.50 to 0.99)	substantial
Inter-grader/observer agreement		
Oivi	0.854 (0.80 to 0.91)	Almost perfect
Topcon NW400	0.891 (0.84 to 0.95)	Almost perfect
	Sensitivity (95% CI)	Specificity (95% CI)
Grader 1- Any DR	94.64% (85.13%-98.88%)	99.73% (98.52%-99.99%)
Grader 1- Referrable DR	90.48% (77.38%-97.34%)	100.00% (99.06%-100%)
Grader 2- Any DR	87.72% (76.32%-94.92%)	99.20% (97.68%-99.83%)
Grader 2- Referrable DR	88.37% (74.92%-96.11%)	100.00% (99.06%-100%)

## References

[1] Lee R, Wong TY, Sabanayagam C (2015). Epidemiology of diabetic retinopathy, diabetic macular edema and related vision loss. Eye Vis (Lond).

[2] Kropp M, Golubnitschaja O, Mazurakova A (2023). Diabetic retinopathy as the leading cause of blindness and early predictor of cascading complications-risks and mitigation. EPMA J.

[3] El-Asrar AM, Al-Rubeaan KA, Al-Amro SA, Moharram OA, Kangave D (2001). Retinopathy as a predictor of other diabetic complications. Int Ophthalmol.

[4] Scanlon PH (2017). The English National Screening Programme for diabetic retinopathy 2003-2016. Acta Diabetol.

[5] Rahmati M, Smith L, Boyer L (2024). Factors affecting global adherence for the uptake of diabetic retinopathy screening: a systematic review and meta-analysis. Am J Ophthalmol.

[6] Sinclair SH (2006). Diabetic retinopathy: the unmet needs for screening and a review of potential solutions. Expert Rev Med Devices.

[7] Abou Taha A, Dinesen S, Vergmann AS, Grauslund J (2024). Present and future screening programs for diabetic retinopathy: a narrative review. Int J Retina Vitreous.

[8] Gadkari SS (2018). Diabetic retinopathy screening: telemedicine, the way to go!. Indian J Ophthalmol.

[9] Williams GA, Scott IU, Haller JA, Maguire AM, Marcus D, McDonald HR (2004). Single-field fundus photography for diabetic retinopathy screening: a report by the American Academy of Ophthalmology. Ophthalmology.

[10] Donner A, Eliasziw M (1992). A goodness-of-fit approach to inference procedures for the kappa statistic: confidence interval construction, significance-testing and sample size estimation. Stat Med.

[11] Raman R, Ramasamy K, Rajalakshmi R, Sivaprasad S, Natarajan S (2021). Diabetic retinopathy screening guidelines in India: All India Ophthalmological Society diabetic retinopathy task force and Vitreoretinal Society of India Consensus Statement. Indian J Ophthalmol.

[12] Nderitu P, do Rio JM, Rasheed R, Raman R, Rajalakshmi R, Bergeles C, Sivaprasad S (2021). Deep learning for gradability classification of handheld, non-mydriatic retinal images. Sci Rep.

[13] Wilkinson CP, Ferris FL 3rd, Klein RE (2003). Proposed international clinical diabetic retinopathy and diabetic macular edema disease severity scales. Ophthalmology.

[14] Li HK, Hubbard LD, Danis RP, Esquivel A, Florez-Arango JF, Krupinski EA (2010). Monoscopic versus stereoscopic retinal photography for grading diabetic retinopathy severity. Invest Ophthalmol Vis Sci.

[15] Vujosevic S, Benetti E, Massignan F (2009). Screening for diabetic retinopathy: 1 and 3 nonmydriatic 45-degree digital fundus photographs vs 7 standard early treatment diabetic retinopathy study fields. Am J Ophthalmol.

[16] Murgatroyd H, Cox A, Ellingford A, Ellis JD, Macewen CJ, Leese GP (2008). Can we predict which patients are at risk of having an ungradeable digital image for screening for diabetic retinopathy?. Eye (Lond).

[17] Suansilpong A, Rawdaree P (2008). Accuracy of single-field nonmydriatic digital fundus image in screening for diabetic retinopathy. J Med Assoc Thai.

[18] Soleimani M, Alipour F, Taghavi Y (2023). Single-field fundus photography for screening of diabetic retinopathy: the prevalence and associated factors in a population-based study. Diabetes Ther.

[19] Murgatroyd H, Ellingford A, Cox A, Binnie M, Ellis JD, MacEwen CJ, Leese GP (2004). Effect of mydriasis and different field strategies on digital image screening of diabetic eye disease. Br J Ophthalmol.

[20] Ku JJ, Landers J, Henderson T, Craig JE (2013). The reliability of single-field fundus photography in screening for diabetic retinopathy: the Central Australian Ocular Health Study. Med J Aust.

[21] Lopez-Bastida J, Cabrera-Lopez F, Serrano-Aguilar P (2007). Sensitivity and specificity of digital retinal imaging for screening diabetic retinopathy. Diabet Med.

[22] Sengupta S, Sindal MD, Besirli CG (2018). Screening for vision-threatening diabetic retinopathy in South India: comparing portable non-mydriatic and standard fundus cameras and clinical exam. Eye (Lond).

[23] Gajiwala UR, Pachchigar S, Patel D, Mistry I, Oza Y, Kundaria D, B R S (2022). Non-mydriatic fundus photography as an alternative to indirect ophthalmoscopy for screening of diabetic retinopathy in community settings: a comparative pilot study in rural and tribal India. BMJ Open.

[24] Piyasena MM, Yip JL, MacLeod D, Kim M, Gudlavalleti VS (2019). Diagnostic test accuracy of diabetic retinopathy screening by physician graders using a hand-held non-mydriatic retinal camera at a tertiary level medical clinic. BMC Ophthalmol.

[25] Abràmoff MD, Lavin PT, Birch M, Shah N, Folk JC (2018). Pivotal trial of an autonomous AI-based diagnostic system for detection of diabetic retinopathy in primary care offices. NPJ Digit Med.

[26] Mead A, Burnett S, Davey C (2001). Diabetic retinal screening in the UK. J R Soc Med.

[27] Grzybowski A, Rao DP, Brona P, Negiloni K, Krzywicki T, Savoy FM (2023). Diagnostic accuracy of automated diabetic retinopathy image assessment softwares: IDx-DR and Medios artificial intelligence. Ophthalmic Res.

[28] Doğan ME, Bilgin AB, Sari R, Bulut M, Akar Y, Aydemir M (2024). Head to head comparison of diagnostic performance of three non-mydriatic cameras for diabetic retinopathy screening with artificial intelligence. Eye (Lond).

[29] Cigolle CT, Blaum CS, Lyu C, Ha J, Kabeto M, Zhong J (2022). Associations of age at diagnosis and duration of diabetes with morbidity and mortality among older adults. JAMA Netw Open.

